# Triggered self-assembly of magnetic nanoparticles

**DOI:** 10.1038/srep23145

**Published:** 2016-03-15

**Authors:** L. Ye, T. Pearson, Y. Cordeau, O. T. Mefford, T. M. Crawford

**Affiliations:** 1 Smart State Center for Experimental Nanoscale Physics and Department of Physics and Astronomy, University of South Carolina, Columbia, SC 29208, USA; 2 Center for Optical Materials Science and Engineering Technologies (COMSET) and Department of Materials Science and Engineering, Clemson University, 161 Sirrine Hall, Clemson, SC 29634 USA; 3 Present Address: MagAssemble LLC, Irmo, SC 29063 USA

## Abstract

Colloidal magnetic nanoparticles are candidates for application in biology, medicine and nanomanufac-turing. Understanding how these particles interact collectively in fluids, especially how they assemble and aggregate under external magnetic fields, is critical for high quality, safe, and reliable deployment of these particles. Here, by applying magnetic forces that vary strongly over the same length scale as the colloidal stabilizing force and then varying this colloidal repulsion, we can trigger self-assembly of these nanoparticles into parallel line patterns on the surface of a disk drive medium. Localized within nanometers of the medium surface, this effect is strongly dependent on the ionic properties of the colloidal fluid but at a level too small to cause bulk colloidal aggregation. We use real-time optical diffraction to monitor the dynamics of self-assembly, detecting local colloidal changes with greatly enhanced sensitivity compared with conventional light scattering. Simulations predict the triggering but not the dynamics, especially at short measurement times. Beyond using spatially-varying magnetic forces to balance interactions and drive assembly in magnetic nanoparticles, future measurements leveraging the sensitivity of this approach could identify novel colloidal effects that impact real-world applications of these nanoparticles.

Magnetic nanoparticles (NPs) can be coated with a variety of functional molecules, colloidally suspended in aqueous fluids, and remotely manipulated with magnetic fields. They have potential to transform biomedicine^1^,^2^,^3^, medical imaging^4^, drug delivery^5^, cancer therapy^6^, cellular manipulation^7^,^8^, and gene transfection^9^. Directed by magnetic field gradients, these NPs can be used as nanoscale building blocks for nanomanufacturing and template-assisted self-assembly of advanced materials^10^,^11^,^12^,^13^. While colloidal NPs are suspended by local forces that change over lengths comparable to the particle diameter, typically 10 nm, the field gradients used for magnetic separation, filtering, or trapping of nano-to-micro scale particles with wires^14^,^15^,^16^, patterned magnetic films^17^,^18^, or domain walls^19^ are effectively constant over many NP diameters.

In contrast, the magnetic fields generated by the bits used to store information in a magnetic disk drive have gradients that vary over 10 nm in length. These fields are emitted from regions on the disk surface that are a fraction of 10 nm, because the Co grains in a composite alloy magnetic medium have diameters ~5 nm and the grain magnetization can be recorded with spatial precision comparable to or less than the grain size^20^,^21^. These gradients reach 1 × 10^7^ T/m within 20 nm of the disk surface and can be used to template assembly of NPs into user designed micro- to macro- scale structures^22^,^23^. Importantly, these gradients decay exponentially with height, changing rapidly near the disk surface. Therefore the magnetic force exerted on a magnetic NP can vary strongly over a single NP diameter, suggesting a unique method for studying the short range stabilizing forces that are used to suspend colloidal NPs. This force is often generated with an electrically charged polymer or surfactant coating on the NP surface that creates a Coulombic repulsion between NPs, known as electrostatic stabilization^24^,^25^,^26^,^27^. This electrostatic force depends strongly on the properties of the surrounding fluid, particularly its ionic strength (IS)^28^,^29^,^30^, and causes NP interactions across multiple length scales^31^,^32^,^33^.

Properties of colloidal NPs including hydrodynamic diameter and degree of aggregation are typically measured with dynamic light scattering (DLS)^34^,^35^,^36^,^37^. Although advanced techniques for measuring complex interactions between NPs have evolved from light scattering to spatially-resolved real-time electron microscopy^38^ and X-Ray techniques^39^, new approaches for measuring how a NP ensemble assembles in real-time, in a 3-dimensional fluid, and across multiple length scales, are needed for improved understanding and ensuring performance in clinical or manufacturing applications. Here, monitoring self-assembly with optical diffraction^40^ as NPs self-assemble onto a grating template recorded on a magnetic disk, we trigger self-assembly by increasing the IS of the fluid, studying the resulting dynamics as a function of both time and IS.

Figure 1a,b show our apparatus and explain the geometry of the magnetically-recorded template. The medium (see **Methods**) is alternately magnetized along the +x and −x directions, with each magnetized region being 750 nm wide and ~ mm long, creating a template that assembles NPs into a diffraction grating with 1333 lines/mm (See Figure S1). Magnetic fields and gradients are strongest at the regions where the medium magnetization is reversed (See Figure S2), called transitions and labeled by “T”s in Fig. 1a,b. A volume of colloid with electrostatically stabilized magnetic NPs (see **Methods**) is introduced into a fluid cell containing the recording medium, as shown in Fig. 1b. Once the fluid is pumped into the cell, the transition magnetic fields and gradients above the medium magnetize and exert locally varying attractive forces on the NPs respectively, assembling them onto the transitions (Fig. 1a)^22^.

We monitor the dynamics of NP assembly by measuring optical diffraction from the grating as it assembles in real time (Fig. 1b). A p-polarized HeNe laser (wavelength *λ* = 632.8 mm, power output = 10 mW and beam diameter ~0.5 mm) is incident on the grating with a ~5° angle (*α*) through a glass window and the 0.5 mm thick colloidal fluid layer. Light is diffracted from the grating with the diffraction angle (*β* in Fig. 1b) according to the diffraction grating equation^41^,


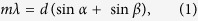


where *m* is the diffraction order and *d* is the grating spacing. 1st order (*m* = +1) diffracted light from the assembling NPs and the light scattered from the NPs in solution are measured with detectors DD and SD in Fig. 1b respectively. These signals are normalized to the incident laser power, yielding diffraction and scattering efficiency (see **Methods** and Supplementary Information Section 3)^42^. Before the NPs are pumped into the fluid cell the diffracted intensity is zero but it increases with time after NPs begin to self-assemble on the disk surface.

Figure 1c compares diffracted and scattered light as a function of time. As IS is increased beyond the base colloid value (IS = 0.0001 M), there is a dramatic enhancement in diffraction efficiency (DE) vs. time (solid black vs. solid red curves in Fig. 1c). We call this enhancement triggered self-assembly. The four dilute NP suspensions with increasing IS (see **Methods**) plotted in Fig. 1c illustrate this effect. From the base colloid to one with IS = 0.031 M, a rapid increase in the slope of DE vs. time is observed, along with an inversion in DE curvature and a 20-fold increase in DE magnitude by 900 s. Increasing the colloidal IS causes a dramatic improvement in the quality of the grating assembled on the template in a given time, as achieving the same efficiency with the base colloid takes 40 times longer than with the triggering colloid (36,000 s vs. 900 s). However, further increasing IS depresses the DE (light and dark blue curves in Fig. 1c), meaning the improvement in grating quality is a sharply peaked function of IS, and the triggering effect occurs in a narrow range of IS values.

For suspensions where peak triggering occurs, there is little change in the light scattered from the fluid cell (the red and black dotted lines in Fig. 1c are right on top of each other). This observation suggests the bulk colloid far from the field gradients is unaffected for the triggering IS values, at least to a level detectable with light scattering. However, for larger IS (IS = 0.056 M and 0.076 M in Fig. 1c), the Scattering Efficiency (SE) does increase with time. In addition, for these large IS suspensions, the DE jumps immediately after NPs are introduced into the fluid cell (initially larger than the triggered DE) but at 200 s crosses under the triggered DE curve and is weaker at long times. This observation suggests that while bulk fluid aggregation happens at larger IS, the aggregates assemble into a lower quality diffraction grating than we obtain with triggered self-assembly. For even larger IS (Fig. 2 and Figure S4), the grating quality at 900 s is comparable to what can be assembled with base suspension NPs at the same time.

In addition to an initial jump, the gratings assembled with IS larger than the peak triggering IS show time-dependent curvature in DE that have inverted again, back to what is seen with the base suspension. This curvature causes these curves (light and dark blue in Fig. 1c) to cross the peak triggering curve near 200 s and each other at 250 s. A simple theoretical calculation of DE vs. time that does not include particle interactions correctly predicts the curvature seen for the base suspension, but not the inverted curvatures seen for IS = 0.031 M (see Supplementary Information, Section **5** for details).

The diffraction vs. time and IS parameter space is presented with more detail in Fig. 2, which shows IS and time dependent dynamics over 900 s for twelve different IS values. Figure 2a plots DE as a colour intensity map vs. time (x-axis) and IS (y-axis). Along the IS axis, three distinct regimes of colloidal stability can be identified (labeled in Fig. 2a by white dashed lines as stable, triggering (semi-stable), and unstable). For the base suspension, the colloid is stable, the DE (self-assembly) increases slowly with time, and the assembly dynamics follow the prediction in Figure S5. In the unstable regime (large IS, increased SE) DE changes weakly, despite larger aggregates producing greater light scattering (see Fig. 1c). However, in the triggering (semi-stable) regime, especially at short times (expanded in Fig. 2b), the DE oscillates with IS, displaying multiple peaks and valleys which are repeatable (see error bars in Fig. 2b). For assembly times between 40 s and 150 s, DE peaks occur at IS = 0.005 M, 0.031 M, 0.056 M and 0.076 M, and valleys occur at IS = 0.015 M, 0.046 M, and 0.066 M. This structure evolves with time, eventually producing a dominant peak at IS = 0.031 M for the longest times measured.

Figure 3a,b compare light scattering and diffraction vs. IS, demonstrating that for small changes in ionic strength, the triggering phenomenon is independent of bulk in-fluid aggregation. As mentioned above the scattered light is independent of time for the base and IS = 0.031 M, finally increasing at larger IS. Because larger particles scatter more light ~ to *b*^6^, where *b* is the particle diameter^43^, an increasing SE would suggest NP aggregation in the colloidal solution. While the scattered light (SE) from our fluid cell starts to increase at smaller IS than seen with DLS that measures the particle hydrodynamic diameter (D_Hydro_) of bulk fluids without the magnetic medium, neither SE nor D_Hydro_ start increasing until IS is greater than 0.031 M, the peak triggering effect shown in Fig. 3b. While larger IS beyond 0.066 M drives in-fluid NP aggregation as expected^24^ (Supplementary Information Section 6), the flat SE and DLS curves at low IS (<0.05 M) suggest that during triggered self-assembly aggregation does not occur over the entire colloid volume. Thus the triggering regime in IS as indicated by two blue dotted lines in Fig. 3b occurs before DLS analysis suggests there is a significant increase in NP hydrodynamic diameter. Figure 3b shows the strongly peaked triggering effect observed at t = 900 s. The peaks seen at IS = 0.005 M and 0.056 M in Fig. 2b remain at long times as shoulders on the sides of the main peak. The minimum at IS = 0.066 M becomes a shoulder at long times as well.

The enhanced DE, constant SE, and decreasing zeta potential in the triggering regime suggest that the bulk colloid has been destabilized, but insufficiently to cause aggregation that we can observe, either with our scattering detector or with DLS. However, within 50 nm of the surface, the magnetic force on a NP increases rapidly, dominating over thermal forces^22^. We propose that triggered self-assembly happens close to the disk surface, requiring a weakly destabilized colloid and a rapidly increasing magnetic force. Since the scattering fluid volume where the triggering occurs a is small fraction of the bulk fluid, the scattering shows little change, while the grating diffraction is strongly enhanced, depending on the force balance between the semi-stable colloid and the transition field gradients.

To better quantify the triggering, particularly the observed DE, we simulate the entire self-assembly process (see **Methods** for simulation details). (i) *N*_0_ (300–10,000) NPs that are initially randomly distributed in a simulation box with a height of 10 *μ*m evolve for a time period of t_*m*_ (5–20 s) to simulate the salt mixing without the assembly template. (ii) These NPs assemble into a grating for a time period of t_*a*_ (10–20 s) under the influence of the recorded field gradient pattern. (iii) A generalized multiparticle Mie (GMM)^44^,^45^ scattering theory is used to calculate DE from the assembled grating for each time step and IS. Key simulated parameters for a grating consisting of *N* assembled NPs are (1) the total number of NPs in the simulation *N*_0_ and (2) their dimensionless spatial phase Φ (see Equation 5 in **Methods**), which are related by


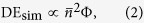


where 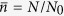. Figure 3c shows simulated 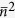 (where 1 is all NPs assembled) and Φ as a function of IS for t_*a*_ = 18 s. 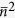 increases rapidly, while Φ decreases with IS as NPs aggregate. This opposite IS dependence produces a peak in DE_sim_ (left axis, solid shapes in Fig. 3d). The right axis in Fig. 3d shows predicted number of NPs per aggregate vs. IS, which increases slowly through the peak in DE_sim_. The IS values used for the simulations (the bottom axis in Fig. 3d) are different from the IS values used for the experiments (bottom axis in Fig. 3b), because the simulation parameters are based on a fixed time step (i.e., Δ*t* in Equation 4 of **Methods**). A fixed (and likely too large) Δ*t* is chosen so that both t_*m*_ and t_*a*_ are in the proper time ranges, and to allow the simulation to complete within a reasonable computation time. We observe the location of the simulated DE peak shifts along the IS axis when Δ*t* is changed, meaning that with a smaller Δ*t* and longer computation time, the simulated peak will shift closer to that observed experimentally, although it is possible that additional parameters are missing from the simulation that shift the experimental IS to larger values. Future studies with fixed IS values require simulations with Δ*t* determined via the aggregation frequency function^46^,^47^ as proposed in Smoluchowski coagulation theory^28^,^48^.

In the simulation NP aggregation strongly affects the observed diffraction. Since magnetic force increases with NP magnetic moment^49^, as NPs begin to aggregate *N* increases rapidly. Similarly, as aggregate size increases, the spatial phase coherence of the grating begins to decrease. This spatial disorder is shown in Fig. 3e–h, which shows simulated NP assembly for 10 gratings lines superposed at the same location. Each panel shows a different IS, increasing left to right, with assembled NPs color coded by aggregate size. For the base solution (Fig. 3e) the simulated grating consists of single NPs and 2–4 NP aggregates, while at large IS (Fig. 3h) the grating consists of aggregates with >8 NPs. The large aggregates produce a grating feature that is wide compared with grating spacing, and this spatial disorder predicts a decrease in DE_sim_ as IS increases.

While the simulation predicts the peak in DE vs. IS, it misses the observed dynamics. It fails to predict the oscillations in DE (Figs 2b and 3b), the inverted assembly curve shape, and the curves crossing each other (Fig. 1c). These discrepancies between experiment and simulation highlight several phenomena in triggered self-assembly that remain unexplained. Importantly, we simulate assembly of NPs within 10 *μ*m of the disk surface, and only predict diffracted signal from the assembled grating. In addition to using shorter time steps, future simulations that include scattering from the colloid at different heights above the surface are needed to identify the origins of triggered self-assembly. Since the experimental fluid height is 50 times larger than the simulated height (0.5 mm vs. 10 *μ*m), such simulations will require significantly greater computational resources than we employ here.

As a final experimental point emphasizing the role of local force balance, the triggering regime depends strongly on the specific salt used. Comparing phosphate buffered saline (PBS) and CaCl_2_ solutions to NaCl, Fig. 4 shows that PBS behaves similarly to NaCl, however without as strong a shoulder at low IS. However the CaCl_2_ is dramatically different, producing larger DE at lower IS in a narrowed triggering regime. The Ca^2+^ ions destabilize colloidal NPs 30 times more effectively than Na^+^ and K^+^ ions for the same IS, in agreement with the Poisson-Boltzmann model prediction that multivalent counterions have a much larger effect on the electrical double layer than monovalent counterions^24^. This effect produces nearly twice the DE at 30x smaller IS than NaCl and PBS. Expanded in Fig. 4 **inset**, the CaCl_2_ peak is 30x smaller in width compared with PBS and NaCl.

In conclusion, we have demonstrated the ability to trigger self-assembly of magnetic nanoparticles and create a better quality nanoparticle diffraction grating over a given time. We perform this triggering by modifying the ionic strength of the colloidal fluid with specific salt compounds, and performing assembly in patterned magnetic field gradients that vary over a nanoparticle diameter. Experimental comparison of diffraction and scattering measurements suggest optical diffraction, combined with triggering, is more sensitive to NP colloidal stability than bulk optical scattering. Simulations of triggered self-assembly based on a Brownian dynamics model suggest both aggregation of particles and spatial phase coherence of assembled particles on the medium surface affect the diffraction signal. However, as the simulations miss several aspects of the observed self-assembly dynamics, additional and more complete simulations are needed to fully understand what causes triggered self-assembly. Our experiments suggest that real-time diffraction, together with NP-sized variations in magnetic force, can detect subtle changes in colloidal stability missed by conventional techniques. More generally, properly designed local magnetic forces, when combined with solution triggering, offer an exciting new means to control and enhance directed self-assembly of NPs.

## Methods

### Magnetic Recording Medium Preparation

**Figure 1:**
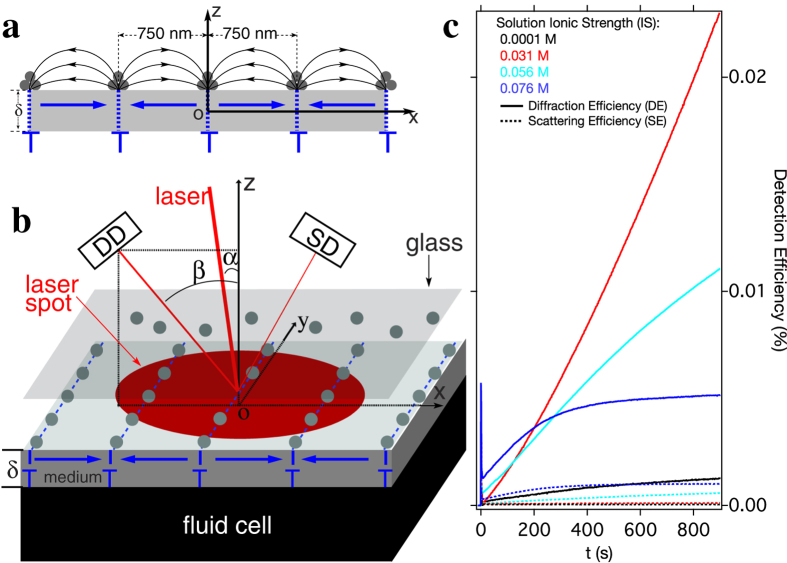
Experimental setup, along with scattering and diffraction vs. time for different ionic strength (IS) suspensions. (**a**) Schematic representation (side view) of a longitudinal magnetic medium recorded with 750 nm spaced transitions and NPs attracted to the transitions (T). The recorded pattern consists of equally-spaced bands (period = 750 nm) that are alternatively configured in north-north or south-south pole orientations. Blue arrows: magnetization orientations. (**b**) Schematic diagram of the fluid cell used to measure scattered and diffracted intensities. DD: diffraction detector, and SD: scattering detector. The 2 × 2 mm^2^ grating is enclosed in a fluid cell with a glass window for optical access. A HeNe laser illuminates multiple grating lines with an incident angle *α*. Photodetectors DD and SD measure the first order diffraction efficiency and static scattering efficiency from the fluid cell respectively. (**c)** The diffraction (scattering) efficiency vs. time for 0.0001 M, 0.031 M, 0.056 M, and 0.076 M IS suspensions. Note the dramatic enhancement in DE compared with SE as IS is increased, followed by a reduction in DE at higher IS.

(1) Commercial longitudinal magnetic media are diced into 1.5 cm diameter circular pieces that are recorded into mm-length stripe patterns with 750 nm spacing between oppositely-magnetized regions using a commercial write/read head (Fig. 1a). The magnetization is oriented along the x-axis, and the magnetization reverses direction over a ~10 nm region of space (determined by the transition parameter *a*)^21^,^23^ (see “T” in Fig. 1a,b). (2) The media templates are then sonicated in Fomblin Perflorosolv (PFS-1 flushing fluid, Solvay Solexis) for 10 minutes to remove the capping layer of hydrocarbon lubricant coated on the surface of these media. (3) The templates are rinsed in methanol for ~10 s and then blow-dried using a N_2_ gun.

### Materials

Commercial ferrofluid, EMG707, is purchased from Ferrotec Inc.(Nashua, NH USA). EMG707 ferrofluid is a water-based colloidal suspension containing Fe_3_O_4_ NPs (nominal particle diameter 10 nm) with a saturation magnetization of 110 Gauss. These EMG707 particles are synthesized via chemical co-precipitation^26^,^27^ using anionic dispersing agents (i.e. surfactants such as oleic acid) to stabilize the NPs against aggregation^24^,^26^,^50^. The phosphate buffered saline solution (PBS) (BDH Chemicals) and cellulose acetate syringe filters (200 nm pore size) are purchased from VWR Inc. (Philadelphia, PA USA). Chemicals including calcium chloride (CaCl_2_) and sodium chloride (NaCl) are purchased from Fisher Scientific (Pittsburgh, PA USA).

### Particle and Suspension Characterization

**Figure 3:**
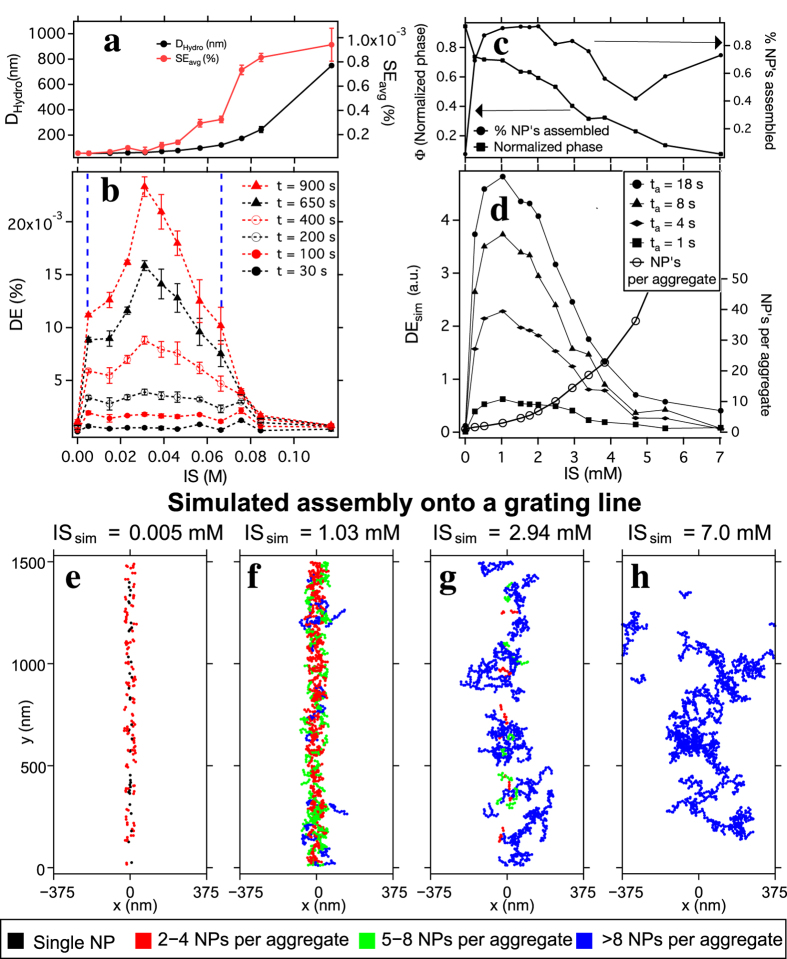
Comparing experimental and simulated self-assembly dynamics. (**a**) Experimental SE from the fluid cell at t = 600 s plotted together with dynamic light scattering (DLS) obtained NP hydrodynamic diameter (D_Hydro_) as a function of IS. Neither SE nor D_Hydro_ increases appreciably until after DE begins to decrease. (**b**) Experimental DE vs. IS to 900 s, showing a strong peak and shoulders in DE that match weaker peaks in Fig. 2. (**c**) Simulated normalized grating spatial phase (left axis) and % NPs assembled (right axis) for 10,000 NPs (*N*_0_ = 10,000). (**d**) Simulated DE vs. IS. The parameters in (**c**) offset and their product yields increasingly larger peaks in DE_sim_ vs. IS as time increases. The right axis shows simulated aggregation vs. IS. Peak DE_sim_ occurs for simulated aggregates consisting of <5 NPs. (**e–h**) Simulated plots of assembled NPs (color coded by aggregate size) at 4 different IS. 10 grating lines are superimposed to show how spatial coherence decreases with increasing IS (the x-axis boundaries are locations of adjacent grating lines).

Transmission electron microscopy (TEM) is used to obtain size distributions for the particle suspensions used in this study. Samples are prepared by dropping diluted nanoparticle suspension onto a copper grid coated with a carbon film. TEM images are acquired at an accelerating voltage of 120 kV on a Hitachi H-7600 microscope. Image analysis is performed using ImageJ software. Approximately 500 particles were measured to have ~15 nm diameter (Figure S1a,b show a TEM image and a TEM-based size distribution of the diameters for our EMG707 NPs). Inductively coupled plasma atomic emission spectroscopy (ICP-AES) (PerkinElmer Optima 3100 RL) is used to determine the amount of iron in the sample. NP hydrodynamic diameter (Fig. 3a) and zeta potential are measured using a Zetasizer NanoZS instrument from Malvern Instruments.

### Particle Suspension Preparation

20 *μ*L of stock EMG707 ferrofluid is diluted with 40 mL of de-ionized water. The dilute suspension is filtered using a cellulose acetate syringe filter. The filtered colloid is referred to as the base suspension in the text. The base suspension has 0.001% volume concentration of NPs, as determined using ICP-AES. To create particle suspensions with higher IS, a small volume (≤200 *μ*L) of salt solution (NaCl, PBS, or CaCl_2_) is mixed with 1 mL of base suspension for 10 s using a vortex mixer. The IS of the mixed solution is calculated as


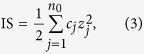


where *n*_0_ is the number of all ions present in the solution, and *c*_*j*_ (*z*_*j*_) is the molar concentration (the charge number) of ion *j*^24^. The base suspension IS is determined from the concentrations of H^+^ and OH^−^ ions as measured with a pH meter.

### Real-time DE and SE Measurements

One hour after making the base suspension, we make real-time measurements with varying IS. After mixing the salt solution with the base suspension, the mixture is immediately pumped into the fluid cell with a syringe pump. The time at which the fluid reaches the laser spot is defined to be t = 0^42^. Both DE and SE curves typically display a spike shortly after t = 0 that remains for 3–5 s (see Fig. 1c), which is caused by laser light scattering from the wave front of the flowing fluid. After acquiring DE and SE data for 900 s, 30 mL of de-ionized water is pumped into the fluid cell with a 100 mL/min flow rate to remove the nanoparticle suspension. Next, the glass slide covering the fluid cell is removed. The template surface is first mechanically cleaned with methanol using a foam-tipped swab that removes previously assembled NPs and then blown dry with N_2_. We ensure the template has been properly cleaned by verifying that the scattering signal from the fluid cell is zero before making the next measurement.

### Numerical Simulation Details

We employ a Brownian dynamics algorithm^48^,^51^ with periodic boundary conditions to account for NP aggregation and Magnetic Field Directed Self-Assembly (MFDSA) dynamics during assembly to produce the simulation results shown in Fig. 3c–h. This algorithm simulates the stochastic force in one dimension (*f*_*stochastic*_) as a Gaussian white noise process^48^,^51^,^52^,^53^, i.e.,


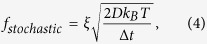


where *k*_*B*_ = 1.38 × 10^−23^ J/K is Boltzmann’s constant, T is the fluid temperature, *D* is the Stokes drag coefficient^52^,^53^, Δ*t* is the simulation time step, and *ξ* is a Gaussian random number with zero mean and unit variance. Several forces determine the ultimate placement of a NP on the medium surface: (1) the magnetic force created by the field gradients of the template (it can be on or off to simulate MFDSA or bulk aggregation respectively), (2) fluid drag, (3) buoyancy, (4) gravity, and (5) interparticle forces that include (i) electrical double layer repulsion, (ii) van der Waals attraction, and (iii) magnetic dipole interactions.

An effective dipole model^54^ is used to calculate NP magnetic moments, and in the simulations, the NPs are presumed to have the same diameter, even as Figure S1 shows they have a distribution of sizes. In addition, the simulation does not allow for changes in aggregate orientation or packing caused by rotation or motion. Finally, beyond total aggregate magnetic moment, no time-dependent spin-spin interactions are included. In addition to simulating assembly of single NPs, the calculation allows NP aggregation to occur when NPs reach a surface-to-surface distance that is ≤*δ*_*c*_ (1 nm). For capture on the surface, a NP or aggregate must reach the surface under the influence of a z-component of the total force that exceeds the weakest z-force at the largest lateral distance that is observed to capture NPs (since the z-component of the magnetic force at a specific height decays with horizontal distance from the transition, i.e. Equation S3 and Figure S2, panel d). This largest distance is experimentally determined from a SEM image of a pattern assembled from base suspension for 12 hours.

Once the simulation of NP trajectories and the arrangement of NPs and aggregates on the surface is complete, as shown in Fig. 3e–h, the generalized multiparticle Mie (GMM) theory^44^,^45^ is used to calculate Φ and DE for *N* assembled NPs as shown in Fig. 3c,d. Φ is computed as


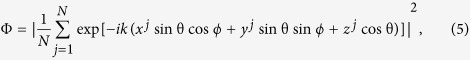


where *i*^2^ = −1, *k* = 2*π*/*λ*, (*x*^*j*^, *y*^*j*^, *z*^*j*^) are Cartesian coordinates of the *j*th NP, and *θ* (*ϕ*) are the polar (azimuthal) angles of the vector from the coordinate origin to the diffraction detector (Fig. 1b). Φ denotes the NP aggregate position relative to the recorded transition, and a larger Φ means the assembled particles are closer to the transitions on average. The simulated DE depends on *N*, Φ, *λ*, as well as NP properties such as size and refractive index. The parallel computation of the simulation was carried out on a cluster consisting of 65 nodes, 12 cores per node, with Intel Xeon 2.8 GHz processors and 24 GB RAM.

## Additional Information

**How to cite this article**: Ye, L. *et al*. Triggered self-assembly of magnetic nanoparticles. *Sci. Rep.* **6**, 23145; doi: 10.1038/srep23145 (2016).

## Supplementary Material

Supplementary Information

## Figures and Tables

**Figure 2:**
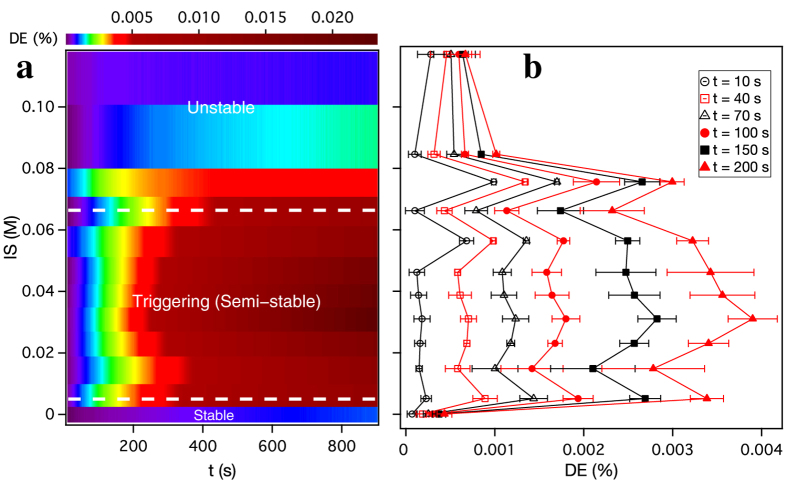
IS dependent self-assembly dynamics at short times for 0.001% volume concentration suspensions. (**a**) Diffraction efficiency (DE, in colour scale) vs. time and IS between 0.0001 M and 0.12 M. For the base suspension, NPs remain fully stable over 900 s with zeta potentials *ζ* ~ −60 mV. The zeta potential is the electrical potential difference between the interfacial double layer of electrostatically stabilized particles and the bulk fluid^24^. The white dotted lines at IS = 0.005 M and 0.066 M mark transitions from stable to semi-stable, and then to unstable. In the semi-stable or triggering regime NPs are weakly destabilized by the added salt, with *ζ* ~ −42 to −52 mV. Increasing IS beyond 0.076 M reduces *ζ* to > −35 mV and causes NP aggregation within the bulk suspension. (**b**) IS vs DE at different times, showing multiple peaks and valleys in the DE depending on the specific IS. Note that a peak appears at IS = 0.031 M for the longest times.

**Figure 4:**
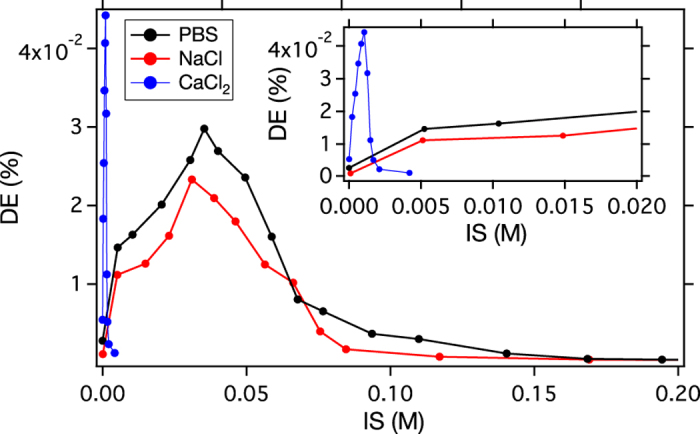
DE vs. IS for PBS, NaCl, and CaCl_2_ at 900 s. PBS contains monovalent counterions (Na^+^ K^+^, and 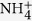), and mono- and divalent co-ions (Cl^−^, 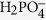, and 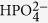). PBS shows IS dependence nearly identical to NaCl, while the CaCl_2_ solution produces a much sharper peak at much smaller IS compared with NaCl and PBS.
