# Supplementary material for: Triggered self-assembly of magnetic nanoparticles
Source: Sci Rep. 2016 Mar 15;6:23145. doi: 10.1038/srep23145 (PMC4791594; doi:10.1038/srep23145)
Supplement: Supplementary Information [file srep23145-s1.pdf]

Supplementary Information:
Triggered self-assembly of magnetic nanoparticles

L. Ye[1,3], T. Pearson[1], Y. Cordeau[2], O.T. Mefford[2], and T. M. Crawford[1]

[1] *Smart State Center for Experimental Nanoscale Physics and Department of Physics and Astronomy, University of South Carolina, Columbia, SC 29208, USA.*

[2] *Center for Optical Materials Science and Engineering Technologies and Department of Materials Science and Engineering, Clemson University, 161 Sirrine Hall, Clemson, SC 29634 USA.*

[3] *Current Address: MagAssemble LLC, Irmo, SC 29063*

1 All-nanoparticle diffraction grating

Figure S1**c** shows a scanning electron microscope (SEM) image of a 750 nm spaced all-nanoparticle diffraction grating that is assembled on the surface of a longitudinal magnetic medium. This medium was produced by removing a portion of a disk drive platter that is recorded with the 750 nm spaced magnetic field template using a dicing saw. This diffraction grating was fabricated by first depositing 0.5 mL of diluted EMG707 ferrofluid with an ionic strength (IS) of 0.031 M on the magnetic medium surface and allowed to remain over the written transitions for 15 min. This colloidal fluid was created by adding the PBS solution to the base suspension. The remaining fluid was removed from the medium surface by slowly tilting the medium. Finally the medium

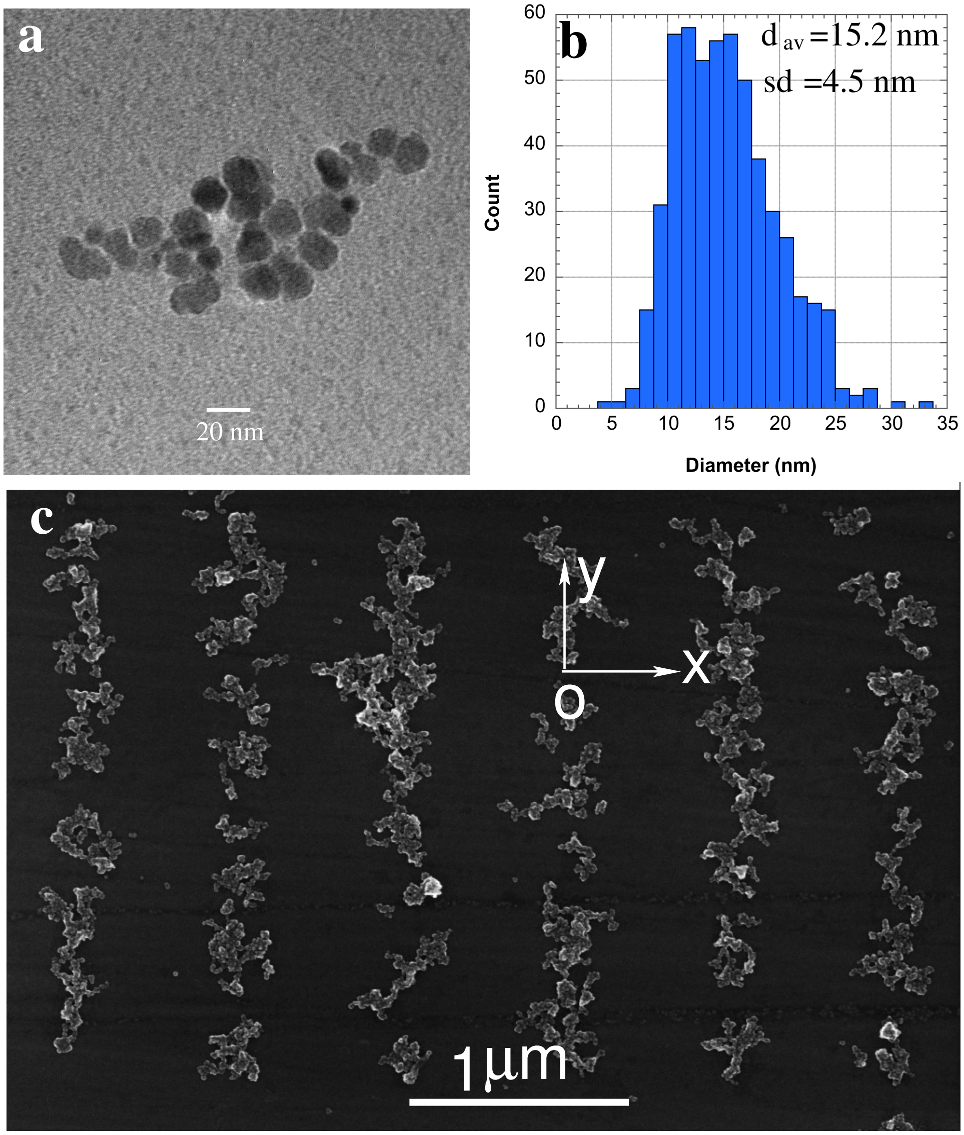

Figure S1: All-nanoparticle diffraction grating assembled from dilute Ferrotec EMG707 ferrofluid. **a**, A TEM image of EMG707 nanoparticles. **b**, Size distribution of EMG707 nanoparticles (d_{av} and sd represent the average particle size and standard deviation of size distribution respectively). **c**, A SEM image of an all-nanoparticle diffraction grating assembled on the disk drive surface.

was immersed into the DI water for 3 s to remove any nanoparticles (NPs) that remain between transitions. Note this procedure is identical to how the assembly is performed in the fluid cell, except for the real-time measurements, the NPs are pumped into the cell with a syringe pump and then rinsed with DI water using a second syringe pump. We have conducted SEM analysis on assembled gratings as a function of IS using the procedure discussed above. The SEM images do not demonstrate the dramatic effects seen in the diffraction data. However we do observe 200 - 1000 nm aggregates that are randomly distributed on the medium for $>$ 0.1 M IS. It is very difficult to reproduce the precise procedure used in the fluid cell and generate coated media suitable for SEM imaging, so the SEM images we have taken are really not directly comparable with the diffraction measurements.

2 Magnetic fields and gradients above transitions on longitudinal disk drive media

The magnetic field above transitions on longitudinal disk drive media can be decomposed into components as

$$\mathbf{H} = \mathrm{H}_x(x,z)\hat{\mathbf{x}} + \mathrm{H}_z(x,z)\hat{\mathbf{z}}. \tag{S1}$$

The field component along $\hat{\mathbf{y}}$ ($\mathrm{H_y}$) is zero because the transition is assumed to be very wide compared with the relevant x- and z- dimensions. Figure S2**a** (S2**b**) shows representative field components $\mathrm{H_x}$ ($\mathrm{H_z}$) as a function of x (z) above a transition in the south-south configuration on longi-

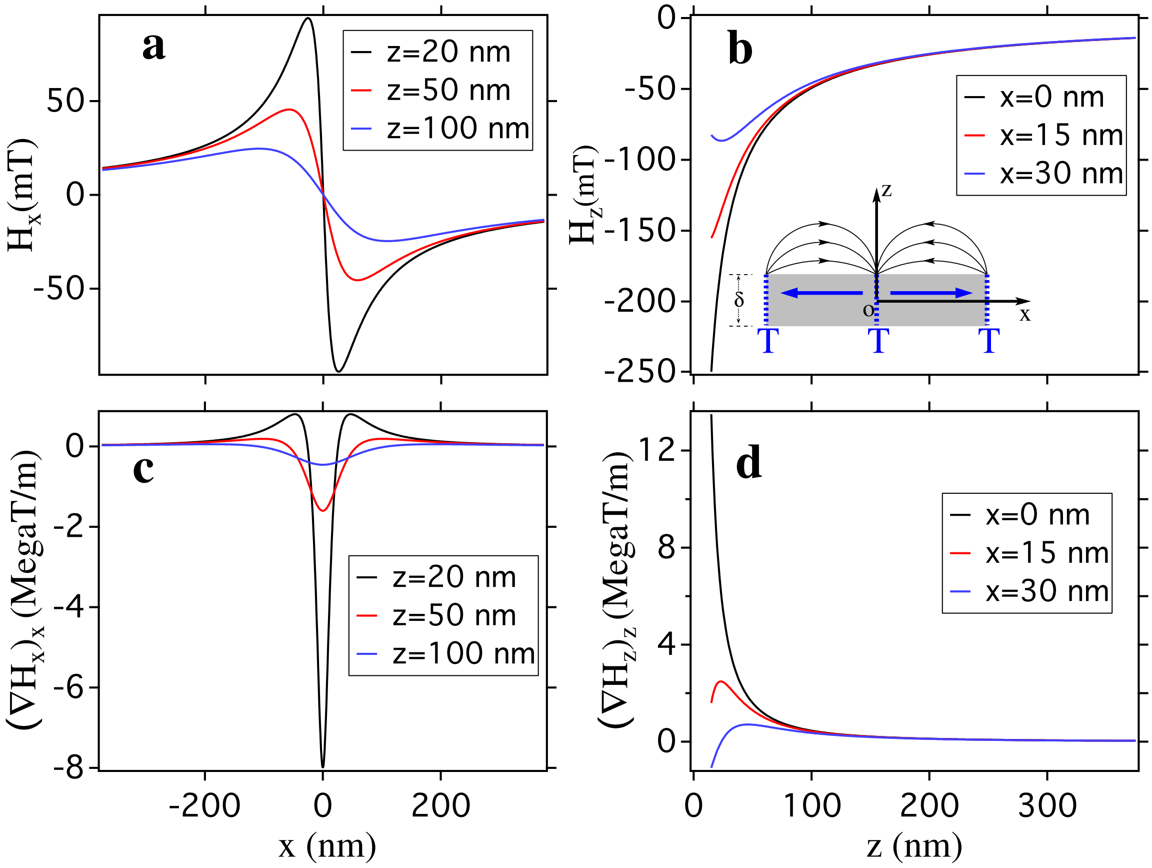

Figure S2: Fields and gradients above transitions with the south-south pole configuration. **a** and **c** show $\mathrm{H_x}$ and $(\nabla \mathrm{H_x})_\mathrm{x}$ as a function of x representively at z = 20, 50, and 100 nm. **b** and **d** show $\mathrm{H_z}$ and $(\nabla \mathrm{H_z})_\mathrm{z}$ as a function of z representively at x = 0, 15, and 30 nm.

tudinal disk drive media (inset to Figure S2**b**). In Figure S2 the coordinate origin is located at the transition and at the center of the medium, with the z axis perpendicular to the medium surface. The field components $\mathrm{H_x}$ and $\mathrm{H_z}$ are calculated using the standard equations for a longitudinally

recorded transition[1],

$$\mathrm{H_x(x,z)} = \frac{M_r}{\pi}\left[\arctan\left(\frac{a+|z|+\delta/2}{x}\right) - \arctan\left(\frac{a+|z|-\delta/2}{x}\right)\right] (|z| \geq \delta/2), \qquad \textbf{(S2)}$$

$$\mathrm{H_z(x,z)} = \pm\frac{M_r}{2\pi}\ln\left[\frac{(a+|z|-\delta/2)^2+x^2}{(a+|z|+\delta/2)^2+x^2}\right] (+ \text{ for } z \geq \delta/2 \text{ and } - \text{ for z} \leq -\delta/2), \qquad \textbf{(S3)}$$

where the medium thickness δ = 30 nm[1], transition parameter a = 10 nm[1], and remanent magnetization, M_r = 4.5 $\times$ 10^5 A/m, as determined using the Vibrating Sampling Magnetometer. The x- and z- directed field gradients of the x- and z- directed fields are also calculated using Eqns. (S2) and (S3).

Figure S2**c** - S2**d** show $(\nabla \mathrm{H_x})_\mathrm{x}$ and $(\nabla \mathrm{H_z})_\mathrm{z}$ as a function of x and z respectively. Importantly, both the field gradient and the gradient of the field gradient are extremely large at the surface of the disk, $\sim 10^7$ Tm^{-1} and 10^{14} Tm^{-2} respectively, but decay dramatically as a function of distance away from the transition. As a result the total field of an array of 750 nm spaced transitions i.e., the summation of contributions from all transitions, is approximately equal to the field from an individual transition. Note the fields and field gradients for the north-north configuration have the same amplitude and opposite sign.

3 Real-time diffraction and scattering measurements

The total laser signal reaching the diffraction detector (DD in Figure 1**b**) includes the scattered signal from the fluid/surface ($>$ 80% of the scattering is from the fluid for the longest measurement time as determined by horizontally translating the grating), and the diffracted intensity from an

assembled grating. The DE from the assembling grating is obtained by subtracting the scattered signal from the total DD signal. The right magnitude to subtract is found by calibrating the relative difference in scattered signal between the DD and the scattering detector (SD in Figure 1**b**). To

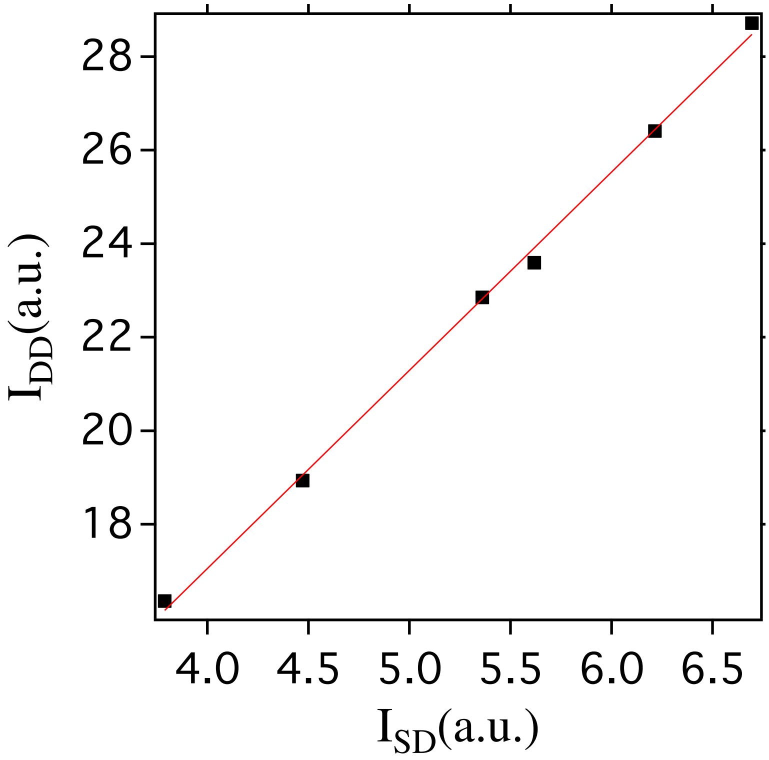

Figure S3: Calibration of the scattered signals on detectors DD (I_{DD}) and SD (I_{SD}).

perform this calibration, a 0.2 mm thick silicon wafer is placed on top of the medium template in the fluid cell, i.e. to avoid any diffraction due to an assembling grating. Thus the DD signal comes entirely from scattered laser light. After pumping a series of ferrofluids with varying concentrations and particle sizes into the cell, the scattered light from the fluid is recorded simultaneously on both DD and SD, and the intensities at 15 minutes are plotted for each ferrofluid as shown in Figure S3. The data from the two detectors are linearly related, and a linear fit yields a conversion factor

= 4.24. Therefore, to obtain the correct scattering value to subtract from the DD, the SD signal is scaled by 4.24 and then subtracted from the DD signal, in order to yield the correct diffracted intensity during real-time assembly.

4 Diffraction and scattering efficiencies for nanoparticle suspensions with large ionic strengths

The solid and dashed curves in Figure S4 show the real-time diffraction efficiency (DE) and scattering efficiency (SE) respectively as a function of time for representative EMG707 NP suspensions with IS $\geq$ 0.076 M. The DE and SE data for IS = 0.076 M IS that are shown in Figure 1**c** are included here for comparison. For IS beyond the triggering range, the total increase in DE by 900 s decreases until the DE no longer increases at all with time for IS = 0.169 M. for values up to IS = 0.118 M, the SE grows with time and the SE at 900 s keeps increasing, which demonstrates that bulk fluid NPs aggregate faster with larger IS beyond the triggering range. For IS = 0.169 M, the SE (like the DE) remains constant in time, suggesting that after the salt mixing process, there is little additional aggregation. Interestingly, the average SE at 0.169 M is smaller than for IS = 0.118 M at long times. One reason for this could be that for IS = 0.169 M, largest aggregates have already sedimented out in the syringe/tubing before they reach the fluid cell, while for IS = 0.118 M additional aggregation occurs in the fluid cell.

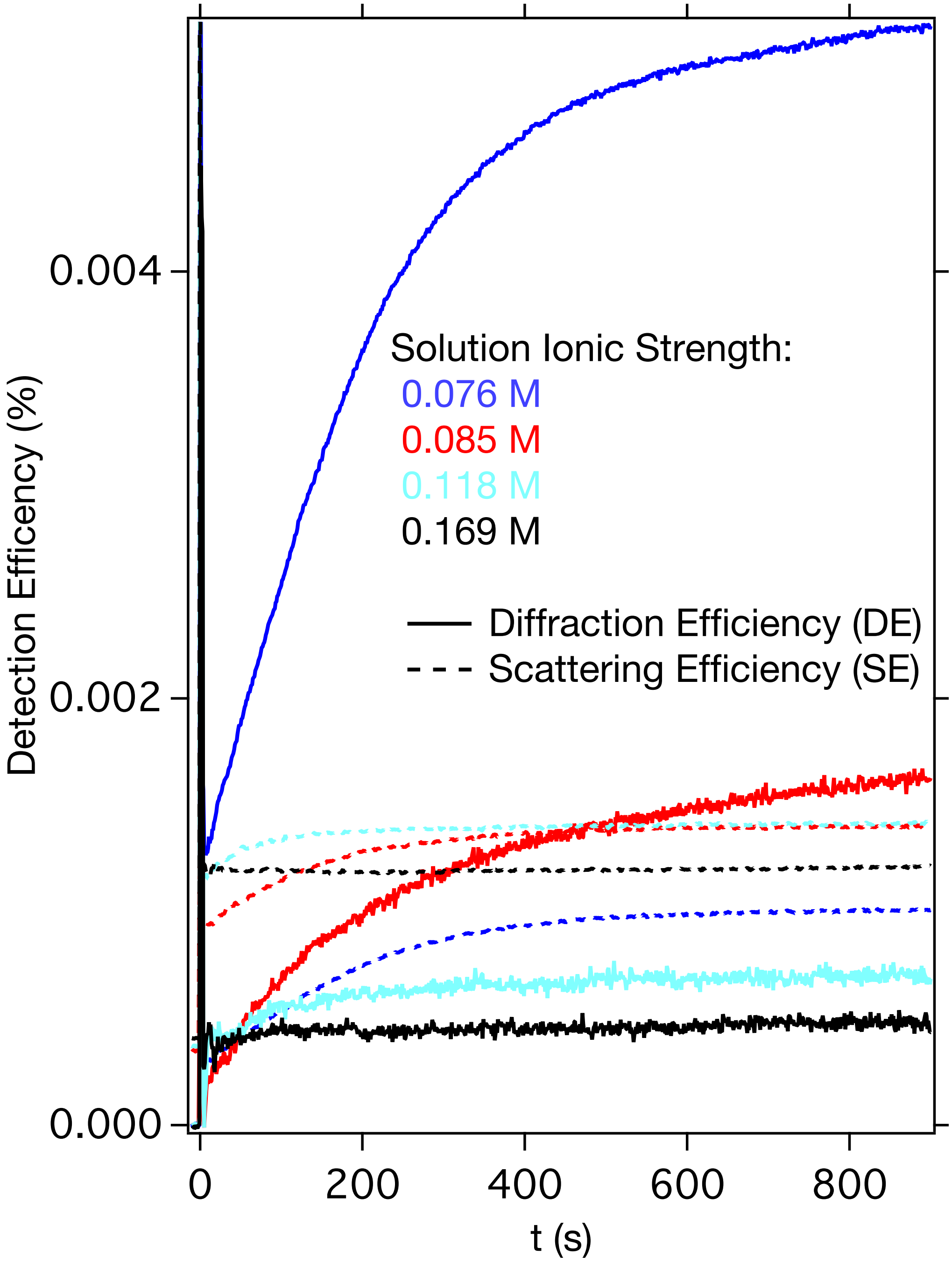

Figure S4: DE (solid) and SE (dashed) vs time for 0.076 M, 0.085 M, 0.0118 M and 0.169 M ionic strength suspensions.

5 Simple theoretical model of nanoparticle self-assembly

In the triggered self-assembly range of IS values, we experimentally observe a change in the curvature of DE as a function of time. Figure S5 shows theoretically calculated diffraction intensities as a function of coating time for varying suspension (13 nm diameter F_3O_4 NPs) concentrations (volume concentrations of NPs: 0.002%, 0.001%, and 0.0005%). The DE in Figure S5 is obtained using a generalized multiparticle Mie (GMM) approach[2,3] for computing the light scattered from assembled NPs that are initially randomly distributed in a hypothetical box above the medium. The placement of the NPs after magnetic field-directed self-assembly is obtained by calculating the trajectory of each NP as directed by the magnetic and the fluid drag force using Newton's second law. This calculation assumes that the nanoarticles do not interact with each other throughout their trajectory toward the disk surface, and it ignores the other interaction forces including the van der Waals attraction and the electrical double layer repulsion that depend on the ionic strength. Therefore, the ionic strength is not included in this simplified calculation. Interestingly, all three concentrations show negative curvatures, in contrast to the positive curvature seen near the triggered DE peak in Figure **1c**.

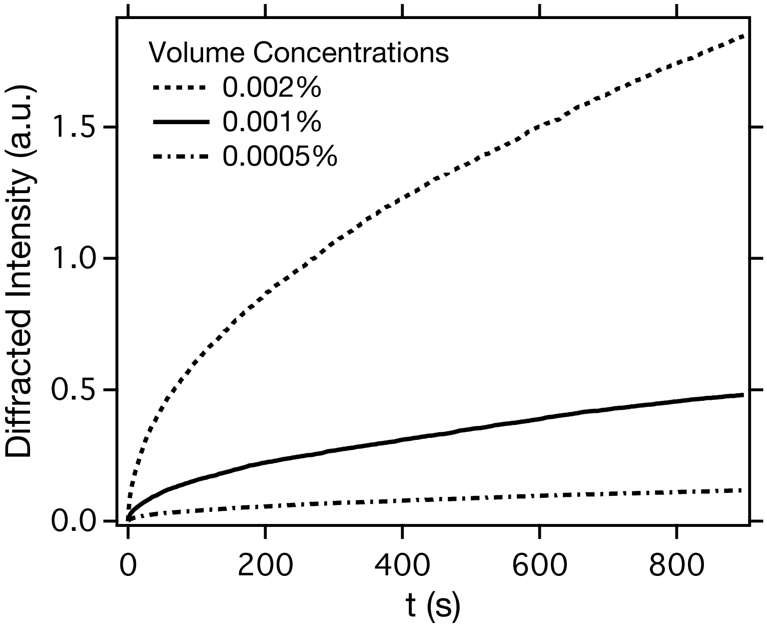

Figure S5: Multiparticle Mie scattering calculation of diffracted intensity vs. time for varying suspension concentrations.

6 Impact of the suspension ionic strength on nanoparticle stability and self-assembly

Colloidally suspended particles undergo Brownian motion and aggregate in liquid due to van der Waals interactions[4–7]. To achieve a stable suspension, the EMG707 particles are coated with anionic surfactants on the particle surface that generate interparticle Coulomb repulsions to prevent particles from aggregation (see Materials in **Methods**). This repulsion arises from the interaction between electrical double layers[8] around the particle surfaces, where charged surfaces interact with electrolyte ions (e.g., added salt ions) in the suspension. The interaction energy (ψ) between

particles i and j is numerically calculated as[9,10]

$$\psi_{ij} = \begin{cases} 4\pi\epsilon\epsilon_0\psi_0^2\dfrac{r_ir_j}{r_i+r_j}\ln[1+\exp(-\kappa\mathrm{H})] & \text{if } \kappa r \geq 5 \\ 4\pi\epsilon\epsilon_0 r_i r_j Y_i Y_j(\dfrac{\mathrm{k_b}T}{e})^2\dfrac{\exp(-\kappa\mathrm{H})}{\mathrm{H}+r_i+r_j} & \text{if } \kappa r < 5, \end{cases} \tag{S4}$$

where ϵ_0 is the permittivity of free space, ϵ is the dielectric constant of water, ψ_0 is the surface potential that is approximately equal to the particle zeta potential ζ, H is the surface-to-surface distance between particles, e is the electrical charge, $\mathrm{k_b}$ is the Boltzmann constant, T is the temperature, and Y is the dimensionless effective surface potential[10–12]. In Eq. (S4), $1/\kappa$ is a characteristic decay length, known as the Debye length, which is defined to be[8]

$$\kappa^{-1} = \sqrt{\frac{\epsilon_0\epsilon_r\mathrm{k_b}T}{2e^2\mathrm{N_A}\mathrm{IS}}} \tag{S5}$$

where $\mathrm{N_A}$ is the Avogadro number.

References

1. Wang, S. X. & Taratorin, A. M. *Magnetic Information Storage Technology: A Volume in the Electromagnetism Series* (Academic press, 1999).

2. Xu, Y.-l. Electromagnetic scattering by an aggregate of spheres: far field. *Applied optics* **36**, 9496–9508 (1997).

3. Xu, Y.-l. & Gustafson, B. Å. A generalized multiparticle mie-solution: further experimental verification. *Journal of Quantitative Spectroscopy and Radiative Transfer* **70**, 395–419 (2001).

4. Rosenzweig, R. E. *Ferrohydrodynamics* (Courier Dover Publications, 1997).

5. Hartley, P., Parfitt, G. & Pollack, L. The role of the van der waals force in the agglomeration of powders containing submicron particles. *Powder technology* **42**, 35–46 (1985).

6. Lalatonne, Y., Richardi, J. & Pileni, M. Van der waals versus dipolar forces controlling mesoscopic organizations of magnetic nanocrystals. *Nature materials* **3**, 121–125 (2004).

7. Herman, M. C. & Papadopoulos, K. D. Effects of asperities on the van der waals and electric double-layer interactions of two parallel flat plates. *Journal of colloid and interface science* **136**, 385–392 (1990).

8. Israelachvili, J. N. *Intermolecular and surface forces: revised third edition* (Academic press, 2011).

9. Hunter, R. Foundations of colloid scienceclarendon press (1987).

10. Liu, H. H., Surawanvijit, S., Rallo, R., Orkoulas, G. & Cohen, Y. Analysis of nanoparticle agglomeration in aqueous suspensions via constant-number monte carlo simulation. *Environmental science & technology* **45**, 9284–9292 (2011).

11. Russel, W., Saville, D. & Schowalter, W. Colloidal dispersions. cambridge monographs on mechanics and applied mathematics (1989).

12. Ohshima, H. Effective surface potential and double-layer interaction of colloidal particles. *Journal of colloid and interface science* **174**, 45–52 (1995).
